# Hyperhomocysteinemia and recurrent carotid stenosis

**DOI:** 10.1186/1471-2261-8-1

**Published:** 2008-01-17

**Authors:** Renata Hillenbrand, Andreas Hillenbrand, Florian Liewald, Julian Zimmermann

**Affiliations:** 1 Department of Vascular and Thoracic Surgery, University of Ulm, Ulm, German; 2 Department of General, Visceral and Transplantation Surgery, University of Ulm, Ulm, Germany; 3 Department of Vascular and Thoracic Surgery, Clinic Esslingen; Esslingen a. N; Germany

## Abstract

**Background:**

Hyperhomocysteinemia has been identified as a potential risk for atherosclerotic disease in epidemiologic studies. This study investigates the impact of elevated serum homocysteine on restenosis after carotid endarterectomy (CEA).

**Methods:**

In a retrospective study, we compared fasting plasma homocysteine levels of 51 patients who developed restenosis during an eight year period after CEA with 45 patients who did not develop restenosis. Restenosis was defined as at least 50% stenosis and was assessed by applying a routine duplex scan follow up investigation. Patients with restenosis were divided into a group with early restenosis (between 3 and 18 months postoperative, a total of 39 patients) and late restenosis (19 and more months; a total of 12 patients).

**Results:**

The groups were controlled for age, sex, and risk factors such as diabetes, nicotine abuse, weight, hypertension, and hyperlipidemia. Patients with restenosis had a significant lower mean homocysteine level (9.11 μmol/L; range: 3.23 μmol/L to 26.49 μmol/L) compared to patients without restenosis (11.01 μmol/L; range: 5.09 μmol/L to 23.29 μmol/L; p = 0.03).

Mean homocysteine level in patients with early restenosis was 8.88 μmol/L (range: 3.23–26.49 μmol/L) and 9.86 μmol/L (range 4.44–19.06 μmol/L) in late restenosis (p = 0.50).

**Conclusion:**

The finding suggests that high plasma homocysteine concentrations do not play a significant role in the development of restenosis following CEA.

## Background

Compared to the best medical therapy, carotid endarterectomy (CEA) or eversions-endarteriectomy has been identified as preferred treatment for symptomatic and asymptomatic patients with high-grade extra cranial carotid stenosis [1].

The long term follow-up of such patients indicates however, that a recurrent stenosis may occur in up to 36% of patients [2], but the incidence of carotid restenosis (CR) is variable and in part dependent on the definition of restenosis and the technique used to calculate its incidence.

Symptomatic or asymptomatic CR is generally attributed to neointimal hyperplasia during the early postoperative period (within 36 months) or recurrent atherosclerosis thereafter [2]. Late CR lesions are indistinguishable from primary atherosclerosis. Neointimal hyperplasia is an intensely studied hyperpalstic reaction in the arterial wall. On gross as well as angiographic or duplex ultrasound interrogation, neointimal hyperplasia is generally smooth in appearance. The exact mechanisms for neointimal hyperplasia are still being investigated, but homocysteine seems to contribute to increased neointimal hyperplasia [3].

Homocysteine is a sulfhydryl amino acid. Its precursor, the essential amino acid methionine, is derived from dietary proteins.

The metabolism of homocysteine stands at the intersection of two pathways: remethylation to methionine, which requires folate and vitamin B12 (or betaine in an alternative reaction); and transsulfuration to cystathionine, which requires pyridoxal-5'-phosphate [4].

Normal levels of Homocysteine are between 5 and 15 μmol/L [5]. There is however, an increased risk for cardiovascular diseases with plasma homocysteine concentrations above 10 μmol/L [6]. Plasma levels between 15 and 30 μmol/L are described as mild hyperhomocysteinemia, levels between 31 and 100 μmol/L are described as moderate and levels above 100 μmol/L are described as severe hyperhomocysteinemia.

Mild hyperhomocysteinemia in fasting conditions can be attributed to any of the following factors: mild impairment in the methylation pathway (i.e. folate or vitamin B12 deficiencies or methylenetetrahydrofolate reductase (MTHFR) thermolability), heterozygous cystathionine beta-synthase defect, vitamin B6 deficiency, or due to smoking, nutritional, hormonal and pharmacological factors [7,8]. In addition, pathologic states (e.g. renal insufficiency, breast, ovarian and pancreatic cancers, and lymphoblastic leukaemia) elevate plasma homocysteine levels [9].

Severe hyperhomocysteinemia is due to rare genetic defects resulting in deficiencies in cystathionine beta synthase, MTHFR, or in enzymes involved in methyl-B12 synthesis and homocysteine methylation [10].

An association between the plasma homocysteine concentration and arteriosclerosis has become the subject of many clinical studies, which have consistently linked moderate hyperhomocysteinemia to symptomatic peripheral vascular, cerebrovascular, and coronary heart disease [11].

The mechanism by which hyperhomocysteinemia causes endothelial damage is not known. Early studies with non-physiological high homocysteine levels showed a variety of deleterious effects on endothelial or smooth muscle cells in culture [12].

The damaging effect may be related to oxidative stress because reactive oxygen species are produced during the auto oxidation of homocysteine [13]. Free radicals induce lipid per oxidation, oxidation of LDL and further intracellular accumulation of cholesterol. Homocysteine increases proliferation of vascular smooth muscle cells and inhibits the growth of endothelial cells [14].

It is the focus of this study to investigate whether there is an association between an elevated plasma homocysteine level and a restenosis after CEA.

## Methods

Routine duplex scan following CEA identified a total of 51 CR patients in the author's hospital (University of Ulm, Department of Vascular Surgery; Germany) that were operated on internal carotid stenosis between 1995 and 2003. As criteria for inclusion in the CR group, patients had at least 50% stenosis but no stenosis within the first three months after operation. 45 patients who did not show CR in a 2 year period after CEA formed a comparative group. All patients were subjected to a postoperative Doppler examination and the postoperative results were recorded. All patients received a thrombocyte aggregation inhibitor or an anticoagulative medication.

Fasting blood samples were taken from each patient at a follow up investigation. Blood was immediately centrifuged (10 minutes, 3000 rpm, 4°C) and plasma samples were stored at -80°C. A fully automated immunoassay for total plasma homocysteine was used to determine the fasting homocysteine level. Furthermore, patients were examined regarding atherosclerosis risk factors (hypertension, diabetes, hypercholesterolemia, nicotine abuse, adiposities) as well as use of medication was recorded.

Approval for the study was obtained from the ethical committee of the University of Ulm. Written informed consent was obtained from all patients before examination.

Patients were subjected to a Duplex examination of the carotid arteries with a high-resolution, real time scanner equipped with a 7.5 MHz imaging transducer, a 4 MHz pulse-wave Doppler transducer, and a 4 MHz continuous-wave transducer.

In the following result section, results are shown as absolute values, and when appropriate as percentage, mean, median, and range. Values between groups are compared through application of a two sided Student's *t*-test. A difference was considered significant at the 0.05 level.

## Results

### A. Patients

The CR group consists of 51 patients of which 33 (65%) were male and 18 (35%) female. Median age was 69 years (range: 50 and 87). With these patients, CR was diagnosed between 3 and 74 months (median 10 months) after CEA.

The comparative group consists of 45 patients of which 38 (84%) were male and 7 (16%) female with a median age of 70 years (range: 50 and 83 years). The postoperative restenosis free period ranged between 24 and 108 months (median 42 months).

Postoperative medication with a thrombocyte aggregation inhibitor or an anticoaguative medication was similar in both groups. In the CR group, 48 out of 51 (94%) patients took acetylsalicylate (Aspirin^®^) or clopidogrel bisulfate (PLAVIX^®^) or both. The remaining 3 (6%) patients took an anticoagulative medication like cumarine (Marcurmar^®^). In the comparative group, 43 out of 45 (96%) patients took thrombocyte aggregation inhibitor. The remaining 2 (4%) patients took anticoagulative medication. Participants of the study were asked for regularly vitamin/folate intake. Only one patient with carotid restenosis took vitamin B regularly.

### B. Restenosis

**Figure 1:**
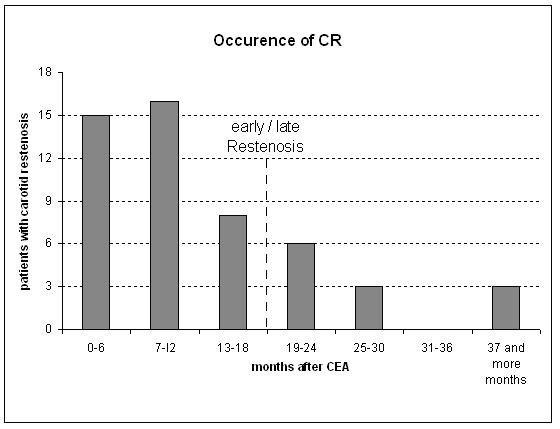
Number of patients with carotid restenosis (CR) according to occurrence in months.

In most cases, CR occurred within the first twelve months (31 out of 51 patients; 61%). After additional 12 months, CR was diagnosed in 45 patients (88%). Only 6 patients (12%) developed CR later than 2 years after CEA. As described above, CR group was divided into a subgroup with early restenosis (3 to 18 months; 39 patients) and a subgroup with late restenosis (19 and more months; 12 patients). This is graphically shown in Figure 1.

### C. Homocysteine concentrations

**Figure 2:**
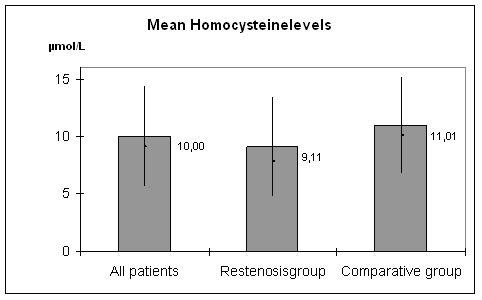
Mean scores of homocysteine levels for all patients, restenosis group, and comparative group.

Mean homocysteine score for homocysteine levels for the entire study group was 10.00 μmol/L whereby levels rage between 3.23 μmol/L and 26.49 μmol/L (median: 9.10 μmol/L, standard deviation: 4.32 μmol/L). The mean score of CR group was 9.11 μmol/L, ranging form 3.23 μmol/L to 26.49 μmol/L (median: 7.78 μmol/L; standard deviation: 4.30 μmol/L). The mean level of comparative group was 11.01 μmol/L (range: 5.09 μmol/L to 23.29 μmol/L; median: 10.08 μmol/L; standard deviation: 4.16 μmol/L; Figure 2). The homocysteine levels in the CR group were significant lower compared to the comparative group (p = 0.03).

### D. Homocysteine concentration and risk factors

Patients were examined regarding five atherosclerosis risk factors (hypertension, diabetes, hypercholesterolemia, nicotine abuse, and adipositas). Examined risk factors were similar distributed in both groups, as exhibited in Table 1. Patients of both groups have 2 risk factors on average (restenosis group 2.0 risk factors; comparative group 2.04 risk factors). Patients in the restenosis group with two or less risk factors have an average homocysteine level of 9.43 μmol/L, patients with three or more risk factors have an average homocysteine level of 8.34 μmol/L (p = 0.41). In the comparative group patients with two or less risk factors have an average homocysteine level of 10.87 μmol/L, patients with three and more have 11.89 μmol/L (p = 0.52). Consequently, there is no difference of homocysteine level in groups with more or less additional risk factors. Likewise no difference was seen comparing count of risk factors in subgroups with homocysteine level below 10 μmol/L and above 10 μmol/L (Restenosis group <10 μmol/L: 2.03 risk factors on average, Restenosis group >10 μmol/L: 2.03 risk factors on average; Control group <10 μmol/L: 2.14 risk factors on average, Control group >10 μmol/L: 1.91 risk factors on average; not significant). The concentrations of total and free plasma homocysteine were higher in active smoker [8]. In our study 8 patients out of 55 with a homocysteine levels below 10 μmol/L smoke, and 7 patients out of 41 with a homocysteine levels above 10 μmol/L smoke. In contrast to literature, there was no significant difference in the homocysteine levels of smokers and non-smokers (9.68 μmol/L, 10.0 μmol/L respectively). The lack of significant difference between homocysteine values between smokers versus non-smokers was probably due to the low patient enrolment. Cross-sectional analysis conducted in recent years confirms the influence of smoking on homocysteine levels. Some studies, however, have failed to find any relationship between smoking and homocysteine levels [15,16]

**Table 1:** Distribution of additional risk factors for restenosis group and comparative group.

**Risk factor**	**Restenosis group**	**Comparative group**
Overweight	7 of 51 (13.7%)	7 of 45 (15.6%)
Diabetes	15 of 51 (29.4%)	14 of 45 (31.1%)
Nicotine	9 of 51 (17.7%)	6 of 45 (13.3%)
Hypertension	42 of 51 (82.4%)	37 of 45 (82.2%)
Hyperlipidemia	29 of 51 (56.9%)	27 of 45 (60.0%)

### E. Homocysteine concentration in early and late CR patients

Patients with an early CR (3 to 18 months) showed a mean homocysteine level of 8.88 μmol/L (range: 3.23 μmol/L to 26.49 μmol/L; median: 7.69 μmol/L; standard deviation: 4.33 μmol/L). Patients with a late CR (more than 18 months after operation) showed a mean homocysteine level of 9.86 μmol/L (range: 4.44 μmol/L to 19.06 μmol/L; median: 9.72 μmol/L; standard deviation: 4.33 μmol/L). Application of t-test showed no significant difference in the homocysteine levels in both groups (p = 0.50).

## Discussion

Data from several studies suggest a mild plasma hyperhomocysteinemia as risk factor for occlusive vascular disease and a 25% risk factor for prevalence of extra cranial carotid artery stenosis in both men and women [17]. The mechanisms by which homocysteine may cause vascular disease include a propensity for thrombosis and impaired thrombolysis, increased production of hydrogen peroxide, endothelial dysfunction and increased oxidation of low-density lipoproteins and Lp(a) lipoproteins [14].

The empirical investigation in this study was concerned with the role of homocysteine in the development of CR. For that purpose, we compared homocysteine levels in patients who had not developed CR 24 months or more after CEA with homocysteine levels in patients who developed CR after three or more months. Our results suggest no systematic relationship between elevated homocysteine levels and the occurrence of CR.

Limitations of our study include the lack of data on serum folate levels, the lack of a genetic analysis regarding MTHFR, and the fact that we measured fasting homocysteine level only once irrespective of transient fluctuations [18]. Further, the patients were sampled between one and four years after CEA and not at the same postoperative time. As with other studies the comparison of results between different labs approved troublesome [19].

Our results are in line with other recent studies that also suggest no systematic relationship between elevated homocysteine levels and the occurrence of CR.

Assadian et al. evaluated the association of plasma homocysteine with early recurrent carotid stenosis following carotid eversion endarterectomy. They found no association of homocysteine with early restenosis [20].

Samson et al. compared homocysteine levels in patients who did not developed CR with those who did within 2 years of CEA. In their study the average homocysteine score for the entire study group was 12.5 μmol/L. The homocysteine values for the subgroups did not differ significantly [21].

Laxdal et al. investigate in a prospective, observational study the effect of elevated serum homocysteine on the likelihood to develop CR after CEA. The authors find a significant association between homocysteine values within the lower two thirds of the normal range and the occurrence of CR [22].

In contrast to that, Southern et al. find in a rat model increasing levels of plasma homocysteine to enhance and accelerated a smooth muscle cell response after CEA leading to increased intimal hyperplasia and luminal stenosis [23]. Furthermore, results of a prospective study by Schnyder indicate that homocysteine levels also can predict long-term outcome after percutaneous coronary intervention regarding target lesion revascularization [24]. On the other hand homocysteine did not appear to influence the outcome of endovascular intervention after endovascular treatment of the above-knee femoro-popliteal artery [25].

As indicated by the studies above there is no consensus yet in literature whether elevated plasma levels of homocysteine are an independent risk factor for restenosis.

Studies on biological properties of human endothelial cells from different types of vasculature and different locations are warranted, specifically with respect to homocysteine metabolism and its effect. A critical question in this context is whether the relationship between homocysteine levels and outcomes after vascular intervention is of causal nature. Plasma homocysteine could also be significantly correlated with age, serum creatinine, and HDL cholesterol, as well as significantly associated with gender and previous MI [24].

## Conclusion

The effects of elevated plasma levels of homocysteine on the rate of CR are contradicting. Our finding suggests that high plasma homocysteine concentrations do not play a significant role in the development of restenosis following CEA. Additional studies are needed to investigate the impact of hyperhomocysteinemia on cardiovascular disease. A critical question in this context is whether the relationship between homocysteine levels and outcomes after vascular intervention is of causal nature. Elevated plasma levels of homocysteine could be a marker, rather than the cause for the respective disease [17].

## Competing interests

The author(s) declare that they have no competing interests.

## Authors' contributions

RH collected the samples and inquired data of patients.

AH performed literature review and performed the statistical analysis.

FL conceived of the study, participated in its design and coordination and helped to draft the manuscript.

JZ performed the duplex scan, conceived of the study, permit of ethical committee, helped to draft the manuscript.

## Pre-publication history

The pre-publication history for this paper can be accessed here:


